# The phenotypic morphology of human lumbar plexus roots associated with changes in the thoracolumbar vertebral count and trade-off

**DOI:** 10.1038/s41598-019-56709-z

**Published:** 2020-01-10

**Authors:** Kaho Ishiguro, Tomokazu Kawashima, Fumi Sato

**Affiliations:** Department of Anatomy, School of Medicine, Toho University, Tokyo, Japan

**Keywords:** Developmental biology, Evolution, Zoology, Anatomy

## Abstract

This study investigated the developmental basis for the human phenotypic morphology of the interaction between the vertebrae and the nerve plexus by evaluating changes in the human lumbar plexus according to various thoracolumbar formulas. The dissection found that the changes in lumbar nerve roots reported by experimental embryology studies to be concomitant with thoracolumbar trade-off, i.e., a change in vertebrae from thoracic to lumbar with no change in the overall thoracolumbar count, were not apparent in humans with the usual 17 or mutant 16 thoracolumbar vertebrae. When vertebral changes in two segments were examined by comparing spines with a reduced thoracolumbar count of 16 to those with an increased count of 18, this tended to show only a single-segment caudal shift of the lumbar plexus. We cannot provide evidence for the phylogenetic difference in the concomitant changes of lumbar nerves and vertebrae, but comparisons between experimental rodents and humans highlighted fewer and shorter lumbar vertebra and more complicated lumbar plexus in humans. Therefore, these multiple differences may contribute to a human phenotypic morphology that is not evident in the concomitant transformation of vertebrae and lumbar nerves reported in experimental rodents.

## Introduction

Lumbosacral transitional vertebrae (LSTV) are congenital vertebral anomalies, such as the sacralization of the fifth lumbar vertebra or lumbarization of the first sacral vertebra. This condition has received attention over many years because of its association with Bertolotti’s syndrome^1–5^. In this syndrome, first described by Bertolotti^6^, the most caudal lumbar transverse process articulates with the ilium in cases of sacralization and/or lumbarization, causing back pain. It is well established that this can cause sacroiliac joint dysfunction and scoliosis^7^.

A radical intervention for this condition is partial bone resection. This requires extensive clinical anatomical knowledge for nerve-sparing surgery^1,4,5^. However, recent advances in imaging analysis using computed tomography (CT) and magnetic resonance imaging have led to reconsideration of how to identify and count the vertebrae and spinal nerves in  LSTV^8–10^, with new debates in spinal surgery about long-standing aspects of anatomy such as the identification of the vertebrae and the evaluation of the spinal nerve segments in vertebral mutations.

Experimental embryology has identified specific interactions between the vertebrae and spinal nerves^11–15^, and studies have reported many new related regulated genes^16–19^. It is expected that more complex mechanisms will be revealed as the analysis continues. However, it is already well established that the Hox genes primarily regulate the development of the vertebrae, spinal nerves, and their interaction^12,15,20^. It is been shown that the same Hox genes are expressed in the same regions in mice, chickens, and humans^20–22^. Interestingly, in Hox 9 and Hox 10 mutant mice with additional thoracic vertebra and a pair of supernumerary ribs, there was a concomitant caudal shift in the lumbar plexus with the transformation of vertebrae and lumbar nerves^23,24^. However, this transformation was reported only in cases where there was a thoracolumbar “trade-off,” i.e., a change in the nature of vertebrae from thoracic to lumbar or vice versa without any change in the overall thoracolumbar count. Thus, it remains to be verified how the lumbosacral plexus is affected when the thoracolumbar count changes. Previous developmental evidence suggests that, when there is a true excess or deficiency in the number of vertebrae, the nerve roots would be expected to shift up or down, respectively, for the concomitant homeotic regulation^23,24^.

The detailed and remarkable anatomical characteristics of the human lumbosacral plexus has been a major subject for analysis in humans with a normal vertebral formula^25–30^. Kumaki (1981, 1994) described transitional changes of the border nerves, focusing on the stratification of the border nerves relative to the three layers of the obliquus abdominis muscles^26,27^. Aizawa (1992) analyzed in detail the branches that comprised the lumbar plexus, including the border nerves, identifying their relative positions within and between nerve segments^25^. Chiba (1992) analyzed the stratified arrangement of the sacral plexus, focusing on the positional relationship relative to the piriformis muscle^28^. Tokita (2006) focused on the branches to the pyramidalis muscle with their surrounding lumbar plexus nerves^30^. These anatomical studies have shown that, even in humans with a typical thoracolumbar count of 17 vertebrae, the lumbosacral plexus exhibits a greater number of individual differences than the brachial plexus that innervates the upper limb muscles.

There have also been anatomical studies of changes in the lumbosacral plexus of humans with anomalous vertebral formulas^31–36^. These reported that the changes in the nerve roots with vertebral mutations were within the range of individual variations in humans with normal vertebral formulas or that the changes were to less than one segment of spinal nerves, including that it was extremely difficult to evaluate the cranio–caudal shift of the nerve roots^31,32,35^. However, these reports compared essentially distinctive cases, including vertebral trade-off cases with normal vertebral counts, such as those with the vertebral formulas 7C–12T–4L–6S (where C refers to cervical vertebrae, T to thoracic, L to lumbar, and S to sacral) and 7C–11T–6L–5S, and those with anomalous total vertebral counts, such as 7C–13T–5L–5S and 7C–12T–6L–5S. For a correct assessment of the interaction between vertebrae and lumbar nerves, it is necessary to analyze these types of cases separately.

Based on the previous reports, no new results on the transformation of human lumbosacral plexus associated with changes in the thoracolumbar mutations can be expected in comparison with the normal number of vertebrae and mutations with a reduced or additional number of vertebrae^31–36^. However, as a result of developmental constraints in vertebral morphogenesis, there have been no reports in the long history of human anatomy that have described increases or decreases in the total number of vertebrae by two or more vertebrae. This suggests that a study strategy for investigating changes in the lumbosacral plexus would be to compare cases with one additional vertebra with those with one less vertebra so that differences related to two vertebrae can be examined.

Based on these considerations, the aims of the present study were (1) to investigate whether the experimental evidence from studies of animals of a caudal shift in the lumbar nerves concomitant with thoracolumbar trade-off also applies to humans with thoracolumbar trade-off, and (2) to investigate whether changes in the thoracolumbar count resulted in transformation of the lumbosacral plexus roots in humans.

## Results

**Figure 1:**
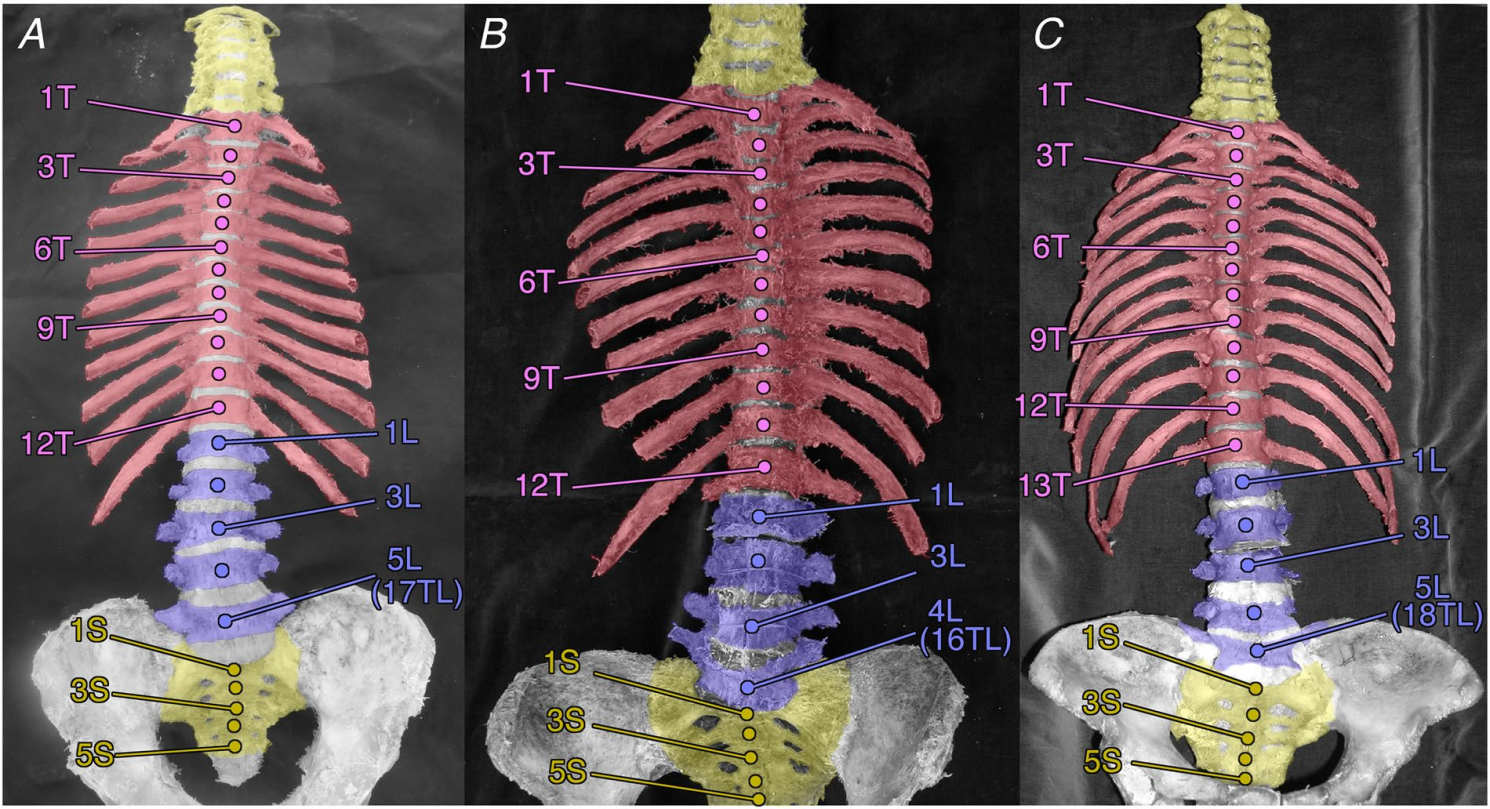
Variation in human thoracolumbar counts. (**A**) Typical, 17 thoracolumbar vertebrae. (**B**) Reduced, 16 thoracolumbar vertebrae. (**C**) Increased, 18 thoracolumbar vertebrae.

In total, 222 sides of 111 cadavers were examined. The typical human vertebral formula (7C–17TL–5S) was observed in 210 sides of 105 cadavers (94.6%, Fig. 1A). Of these, four sides in two cadavers exhibited the thoracolumbar trade-off (7C–11T–6L–5S). In addition, total vertebral number was the same as the typical vertebral formula, but one cadaver exhibited the lumbosacral trade-off (7C–12T–4L–6S, 0.9%). The remaining 10 sides of five cadavers (4.5%) showed mutations in the axial vertebral count. All 10 sides exhibited 7 cervical and 5 sacral vertebrae, and so all these mutations were only thoracolumbar mutations in count: either a reduced thoracolumbar count (7C–16TL–5S), which was observed in eight sides of four cadavers (3.6%, Fig. 1B), or an additional thoracolumbar count (7C–18TL–5S), observed in both sides of one cadaver (0.9%, Fig. 1C).

The entire lumbosacral plexus was dissected, but the present study focused mainly on the nerve branches derived from one or two segments to identify obvious changes between vertebral segments.

### Variability of the nerve roots in the typical 17 thoracolumbar count

**Figure 2:**
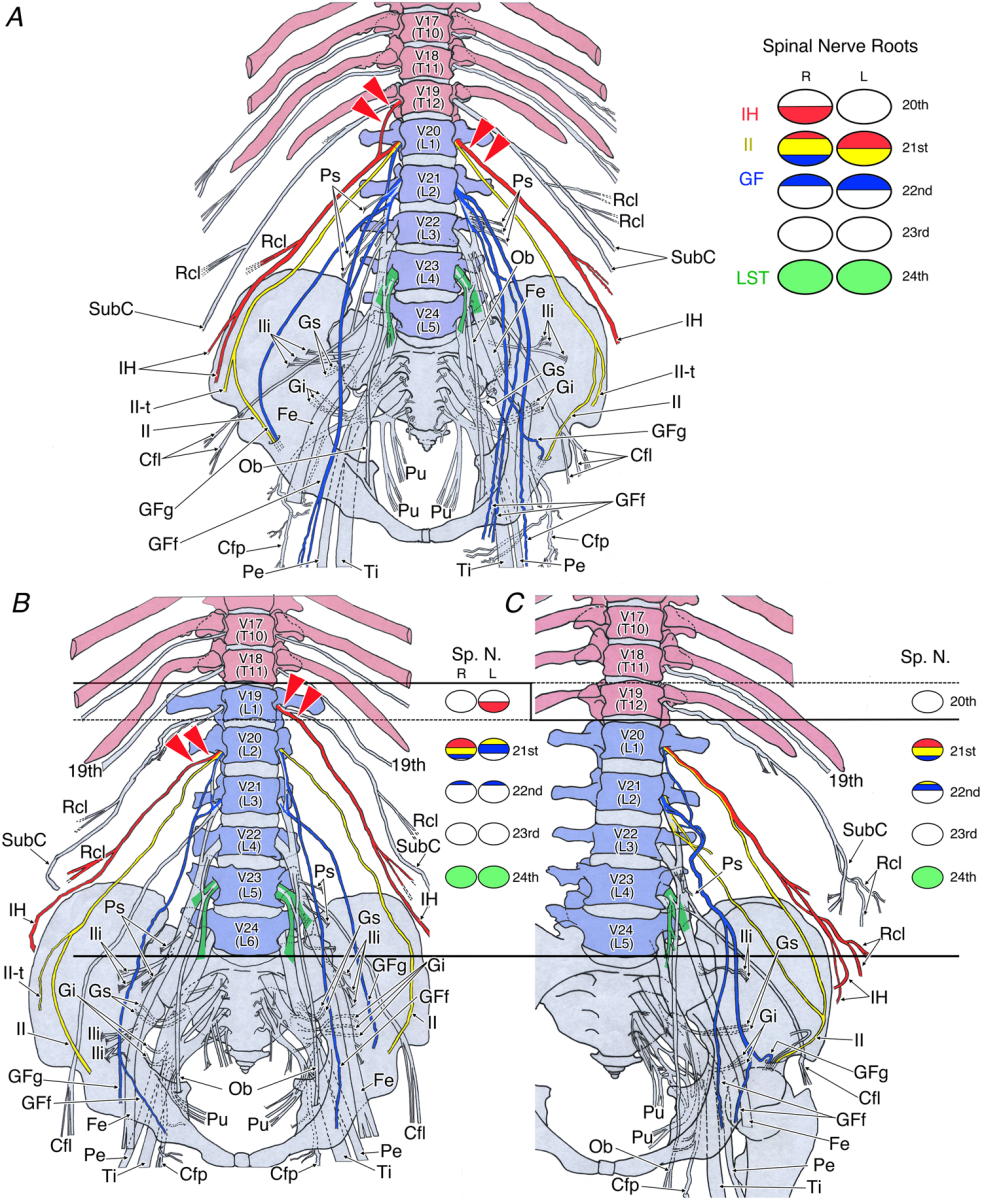
The human lumbosacral plexus in the typical 17 thoracolumbar vertebrae. The circles on the right show the nerve roots of the lumbar plexus. (**A**) A case of typical human lumbar plexus roots in the most common thoracolumbar formula (7C–12T–5L–5S) exhibiting different IH nerve roots on the left and right, as shown by red arrowheads. (**B**,**C**) Changes in the human lumbar plexus involving thoracolumbar trade-off in spines with 17 thoracolumbar vertebrae: (**B**) 11 thoracic and 6 lumbar vertebrae. (**C**) 12 thoracic and 5 lumbar vertebrae. At the time of the caudal shift of the thoracic vertebra, the upper limit of the human lumbar plexus was not always a concomitant change and was unclear for individual variation. Abbreviations: Cfl, lateral femoral cutaneous nerve; Cfp, posterior femoral cutaneous nerve; Fe, femoral nerve; GFf, femoral branch of the genitofemoral nerve; GFg, genital branch of the genitofemoral nerve; Gi, inferior gluteal nerve; Gs, superior gluteal nerve; IH, iliohypogastric nerve; II, ilioinguinal nerve; II-t, transitional ilioinguinal nerve; Ili, branch to the iliacus muscle; LST, lumbosacral trunk; Ob, obturator nerve; Pe, common peroneal nerve; Ps, branch to the psoas major muscle; Pu, pudendal nerve; Rcl, lateral cutaneous branch; SubC, subcostal nerve; Ti, tibial nerve; V16–V23, 16th to 23th vertebra.

In the typical human vertebral formula (7C–17TL–5S), detailed individual variation of the nerve roots in the lumbar plexus was randomly selected and observed as a control in 26 sides of 13 cadavers (Fig. 2).

The same individual often displayed asymmetry in the height of the nerve roots composing the lumbar plexus (Fig. 2A,B). Specifically, the iliohypogastric (IH), ilioinguinal (II), and genitofemoral (GF) nerves were asymmetrical with frequencies of 3/13, 2/13, and 5/13 cadavers, respectively. These findings clearly demonstrated the necessity of data presentation on each side rather than individual data.

In the 26 sides with a thoracolumbar count of the typical 17, the composition of the nerves was as follows:

IH nerve, 20th spinal nerve (10/26 sides), 20–21st spinal nerves (1/26 sides), or 21st spinal nerve (15/26 sides);

II nerve, 20–21st spinal nerves (1/26 sides), 21st spinal nerve (23/26 sides), or 21–22nd spinal nerves (2/26 (sides);

GF nerve, 21st spinal nerve (1/26 sides), 21–22nd spinal nerves (19/26 sides), 22nd spinal nerve (3/26 sides), 21–23rd spinal nerve (1/26 sides), or 22–23rd spinal nerve (2/26 sides);

Lumbosacral trunk (LST), 24th spinal nerve (26/26 sides).

These findings show that the IH, II, and GF nerves exhibited individual variation in the height of roots and sides, whereas the LST were consistent in all examined cases of 17 thoracolumbar counts.

### Transformation in the upper and lower limits of the human lumbar plexus with thoracolumbar trade-off

#### Trade-off with a thoracolumbar count of 16

**Figure 3:**
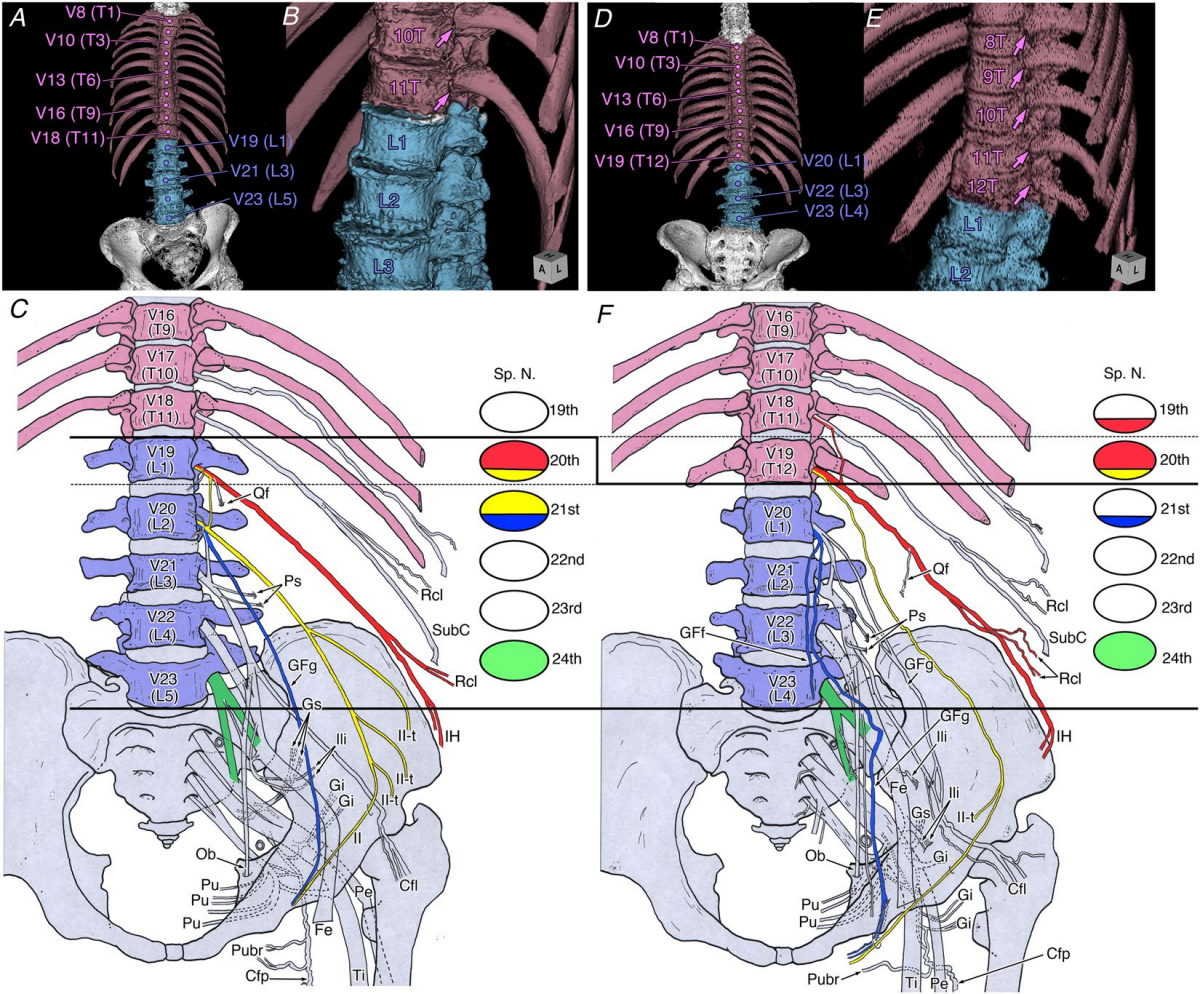
Changes in the human lumbar plexus involving thoracolumbar trade-off in spines with 16 thoracolumbar vertebrae: (**A**–**C**) 11 thoracic and 5 lumbar vertebrae; (**D**–**F**) 12 thoracic and 4 lumbar vertebrae. The circles on the right show the nerve roots of the lumbar plexus. At the time of the caudal shift of the thoracic vertebra, the upper limit of the lumbar plexus underwent a cranial shift in all the sides examined. All our findings of the converse and cranial shift of the anterior (superior) limit of the human lumbar plexus with the thoracolumbar trade-off are contradicted with concomitant transformation reported by studies of experimental embryology. Abbreviations: Cfl, lateral femoral cutaneous nerve; Cfp, posterior femoral cutaneous nerve; Fe, femoral nerve; GFf, femoral branch of the genitofemoral nerve; GFg, genital branch of the genitofemoral nerve; Gi, inferior gluteal nerve; Gs, superior gluteal nerve; IH, iliohypogastric nerve; II, ilioinguinal nerve; II-t, transitional ilioinguinal nerve; Ili, branch to the iliacus muscle; Ob, obturator nerve; Pe, common peroneal nerve; Ps, branch to the psoas major muscle; Pu, pudendal nerve; Pubr, pudendal branch of the posterior femoral cutaneous nerve; Qf, branch to the quadratus lumborum muscle; Rcl, lateral cutaneous branch; Sp. N., spinal nerve; SubC, subcostal nerve; Ti, tibial nerve; V16–V23, 16th to 23th vertebra.

A thoracolumbar count of 16 was observed in eight of the 222 sides (3.6%). These Included 11T–5 L in six sides and 12T–4 L in two sides. In the former, the IH nerves were derived from the 20th spinal nerve; in the latter, they were derived from the 19th–20th spinal nerves in all sides. These findings showed that the caudal trade-off of the lumbar to thoracic vertebra, such as an additional thoracic vertebra with a pair of supernumerary ribs; conversely, the upper limit of the lumbar plexus nerve root exhibited a cranial shift in all the sides examined (Fig. 3).

In the six sides with 11T–5 L, the LST, which forms the lower limit of the lumbar plexus and the boundary between the lumbar and sacral plexuses, was derived from the 23rd spinal nerve (two sides) or the 24th spinal nerve (four sides); in the two sides with 12T–4 L, the LST was derived from the 24th spinal nerve. With the caudal trade-off of a lumbar to a thoracic vertebra, the change in the lower limit of the lumbar plexus root was unclear because it had the same origin or caudal shift.

#### Trade-off with a thoracolumbar count of 17

A thoracolumbar count of 17 was observed in 210 sides (94.6%), of which four sides (1.9%) had the 11T–6 L mutation. The IH nerve, which is the upper limit of the lumbar plexus, was derived from the 20th spinal nerve in three sides and from the 21st spinal nerve in one side (Fig. 2B). The LST was derived from the 24th spinal nerve in all four sides.

In the 12T-6L of the typical 17 thoracolumbar count, the IH nerve was derived from the 20th spinal nerve in 8 out of 22 sides or from the 21st spinal nerve in 14 sides; however, the LST was derived from the 24th spinal nerve in all 22 sides (Fig. 2C).

### Transformation of the human lumbar plexus with different thoracolumbar counts

#### A reduced thoracolumbar count of 16

In the eight sides with a thoracolumbar count of 16, the composition of the nerves was as follows:

IH nerve, 19–20th spinal nerves (2/8 sides), 20th spinal nerve (6/8 sides);

ilioinguinal (II) nerve, 20th spinal nerve (4/8 sides), 20th –21st spinal nerves (1/8 sides) or 21st spinal nerve (3/8 sides);

genital branch of the genitofemoral (GFi) nerve, 20th spinal nerve (1/8 sides), 20th–21st spinal nerves (1/8 sides), 21st spinal nerve (4/8 sides), or 21st–22nd spinal nerves (2/8 sides);

femoral branch of the GF nerve (GFf), 20th spinal nerve (2/8 sides), 21st spinal nerve (3/8 sides), 21st–22nd spinal nerves (2/8 sides) or unknown (1/8 sides);

LST: 23rd spinal nerve (2/8 sides) or 24th spinal nerve (6/8 sides).

#### An increased thoracolumbar count of 18

A thoracolumbar count of 18 was observed in both sides of a single cadaver, but it exhibited asymmetry in height of the nerve roots. The actual composition of each nerve was as follows;

IH, 21st–22nd spinal nerves (1/2 sides) or 22nd spinal nerve (1/2 sides);

II, 22nd spinal nerve (2/2 sides);

GFi, 22nd spinal nerve (1/2 sides) or 22nd–23rd spinal nerve (1/2 sides);

GFf, 22nd–23rd spinal nerves (2/2 sides);

LST, 25th spinal nerve (2/2 sides).

**Figure 4:**
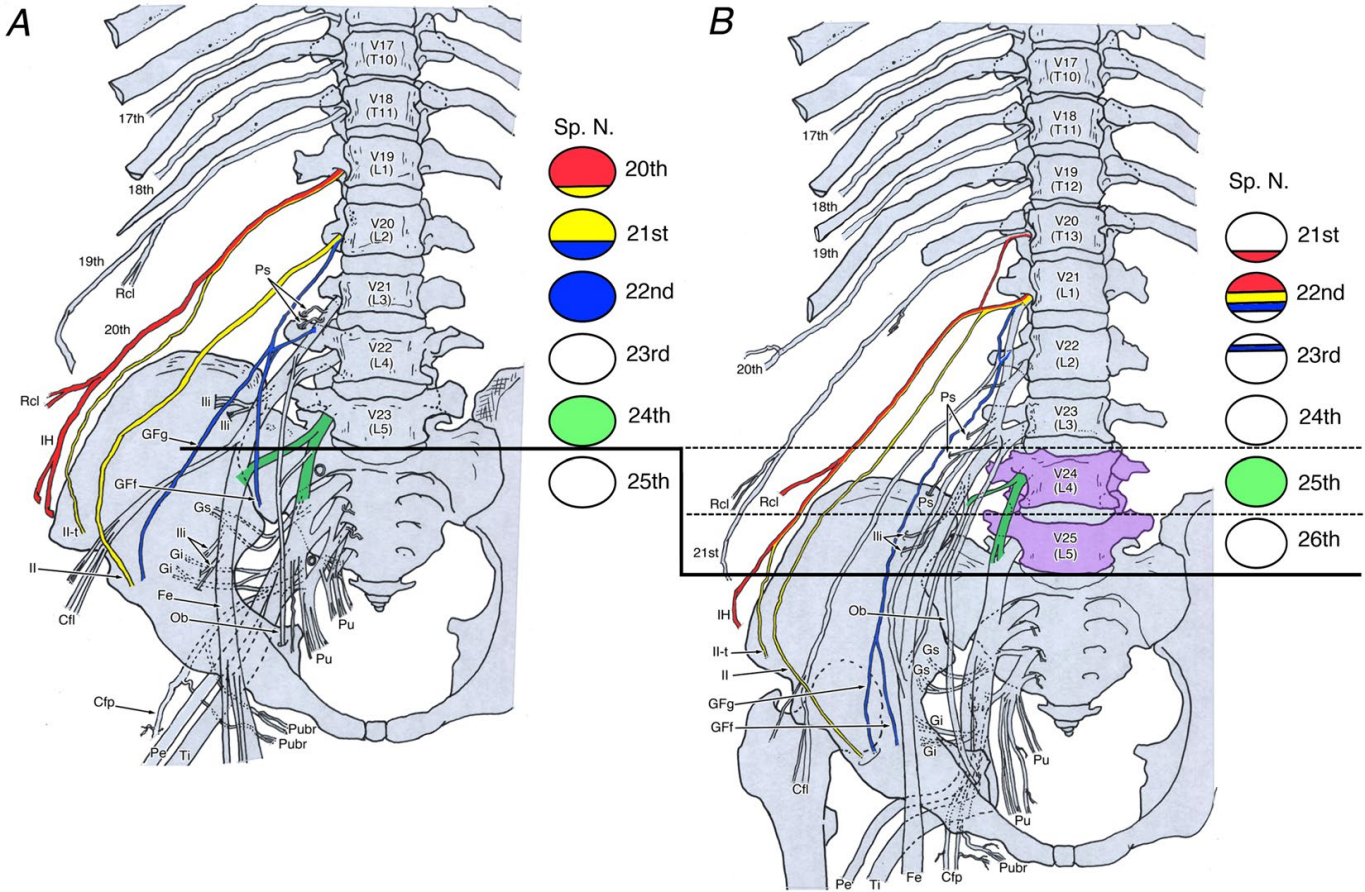
Changes of the lumbosacral plexus roots resulting from different thoracolumbar counts in spines with (**A**) 16 thoracolumbar vertebrae and (**B**) 18 thoracolumbar vertebrae. The circles on the right show the nerve roots of the lumbar plexus. In humans, vertebral changes in two segments results in a shift in nerve roots in only one segment. Abbreviations: Cfl, lateral femoral cutaneous nerve; Cfp, posterior femoral cutaneous nerve; Fe, femoral nerve; GFf, femoral branch of the genitofemoral nerve; GFg, genital branch of the genitofemoral nerve; Gi, inferior gluteal nerve; Gs, superior gluteal nerve; IH, iliohypogastric nerve; II, ilioinguinal nerve; II-t, transitional ilioinguinal nerve; Ili, branch to the Iliacus muscle; Ob, obturator nerve; Pe, common peroneal nerve; Ps, branch to the psoas major muscle; Pu, pudendal nerve; Pubr, pudendal branch of the posterior femoral cutaneous nerve; Qf, branch to the quadratus lumborum muscle; Rcl, lateral cutaneous branch; Sp. N., spinal nerve; SubC, subcostal nerve; Ti, tibial nerve; V17–V25, 17th to 25th vertebra.

**Figure 5:**
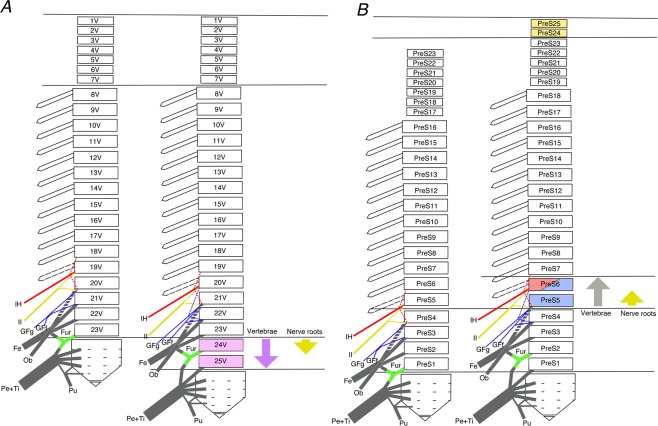
Schematic representations showing the phenotypic changes to the human lumbar plexus associated with different thoracolumbar counts (16 to 18). (**A**) cranial count. In humans, the caudal shift of two segments of thoracolumbar vertebrae results in a caudal shift in nerve roots in only one segment. (**B**) presacral caudal count. Because of the different segmental position of the lumbosacral trunk counting presacral vertebrae as prescral numbers, the positional information about it being near the sacroiliac joint might not have an influence. Abbreviations: Fe, femoral nerve; Fur, furcal nerve or lumbosacral trunk; GFf, femoral branch of the genitofemoral nerve; GFg, genital branch of the genitofemoral nerve; IH, iliohypogastric nerve; II, ilioinguinal nerve; Ob, obturator nerve; Pe, common peroneal nerve; Pu, pudendal nerve; Ti, tibial nerve; 1V–25 V, 1st to 25th vertebra.

Thus, compared with the lumbar plexus in the cadavers with 16 and 18 thoracolumbar vertebrae, all the examined nerves with a thoracolumbar count of 18 showed a caudal shift by 0.5–1 segment when cranial counting the nerve roots from the skull base (Figs. 4 and 5A) and a cranial shift of 1 segment when caudal counting the nerve roots from the sacrum (Fig. 5B).

### Proportion of the human thoracolumbar vertebrae

**Figure 6:**
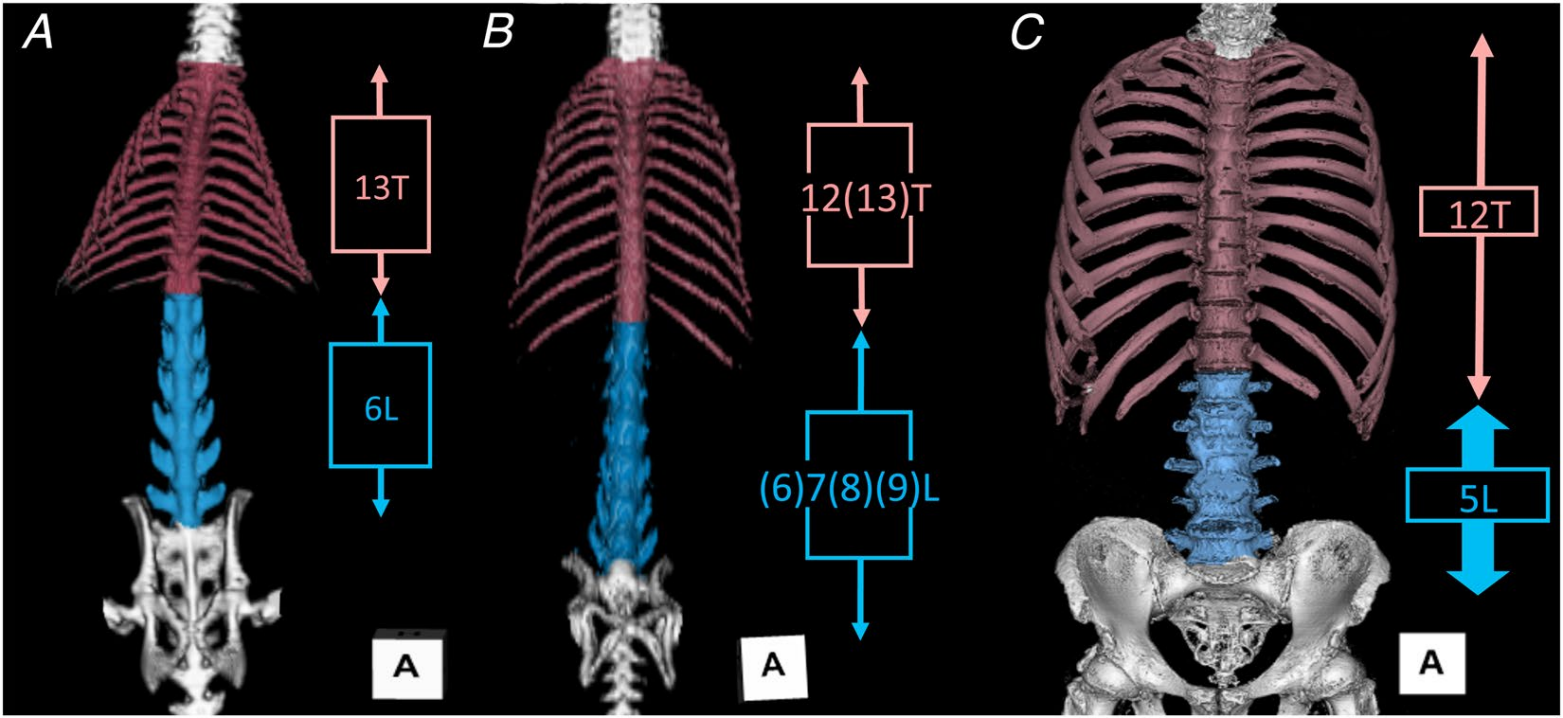
Comparative osteology of the thoracolumbar vertebrae in (**A**) rats, (**B**) flying squirrels, and (**C**) humans. The rats showed no vertebral mutation, even in wild species (**A**), whereas greater vertebral formula variability was observed in the flying squirrels (**B**), an example of a mammal with specialized locomotion (gliding). The vertebral formula was relatively stable in humans (**C**); however, a few exhibited an exceptional thoracolumbar count, with a shorter relative length of the lumbar vertebrae and compression of individual lumbar vertebrae.

In order to obtain clues regarding the unclear relationship between mammalian developmental evidence and the human phenotypic morphology of the lumbar plexus in vertebral mutations, the human vertebral characteristics of proportion and variation were examined. The 110 human cadavers except one case of the lumbosacral trade-off also showed a slight variation in the thoracolumbar count: 16 in 4 (3.6%), 17 in 105 (94.6%), and 18 in 1 (0.9%). Of those with a thoracolumbar count of 17, the most common formula was 12T–5 L (103/105 bodies, 98.1%; Fig. 6C). In osteological analysis using dry bones with most common formula (12T-5L), the total length of the lumbar vertebrae relative to the total length of the thoracic vertebrae was 35.2% ± 1.3% (n = 17).

## Discussion

Studies in physical anthropology and anatomy have described the various patterns of vertebral mutations and its nervous transformation without sex distinction^31–36^. In recent years, it has been reported that humans demonstrate sexual dimorphism in some characteristics of the spinal cord^37–40^, therefore it is preferable to check the influence of sexual changes in vertebral mutations and spinal nerve roots. Surprisingly, all the mutations (16 or 18 thoracolumbar counts) in this study were found in females. In the future, meta-analysis is required to confirm sexual dimorphism in vertebral mutations.

It is well established that the genes Hox 7 to 10 regulate both the thoracolumbar vertebrae and the spinal nerves in rats, chicks, and humans^12,15,20,22^. Hox 10 also plays an important role in regulating the anterior (upper) limit of the lumbosacral plexus roots. Hox 10 knockout mice have been shown to exhibit a caudal shift in the anterior limit of the lumbosacral plexus due to the formation of an additional rib; however, there has been no evidence of this in other mammals, including humans^20,22,23^.

In this study, therefore, we revisited the transformation of the human lumbosacral plexus observed with thoracolumbar mutations involving trade-offs or changes in the thoracolumbar count to establish the phenotypic characteristics of humans. Various individual variation in the normal human vertebral formula have previously been reported^25–28^ as described in the present study, and there have been several reports of changes observed by comparing the lumbosacral plexus between normal spines and those with vertebral anomalies, although no clear features have been identified^31–36^. This lack of clear features may have been because no distinction was made in the previous studies between the cases with mutations in the thoracolumbar count and those in which the mutation was a trade-off. In the present study, we classified the examined cadavers into two groups and then attempted to identify their phenotypic changes. Furthermore, we investigated the transformation of the lumbar plexus, which had not been observed in conventional comparisons between normal spines and those with a single anomalous vertebral mutation, through an analysis that compared the vertebrae in the two segments.

Developmental studies have reported that the anterior (upper) limit of the lumbosacral plexus shifted caudally by an additional rib in Hox 9 and 10 knockout mice^23,24^. In the present study, the thoracolumbar trade-off in humans with a thoracolumbar count of 17 was unclear because it was within the range of the individual variation in the normal human lumbar plexus, as previously reported. Conversely, our findings for those with a thoracolumbar count of 16 clearly contradicted the developmental evidence obtained from mice. In four cases of 11 thoracic and 5 lumbar vertebrae of the standard vertebral formula with a thoracolumbar count of 16, IH was the 19th spinal nerve. In two cases with 12 thoracic and 4 lumbar vertebrae, IH was the 18th–19th spinal nerves. Thus, surprisingly, the anterior (upper) limit of the lumbar plexus had shifted cranially by 0.5–1 segment without a caudal shift. The lumbosacral trunk, which is the posterior (lower) limit of the lumbar plexus, was mostly the 24th spinal nerve. This suggests that it may be significant to consider it as a shorter or longer trunk type, which are a fewer or an additional transitional nerve components from the abdominal wall to the lower limbs^26,27^, or the position of the LST, which is the boundary between the lumbar and sacral plexuses, may be determined by its relative position to the surrounding structures, such as the pelvis or sacroiliac joint, as research objectives in previous anatomical studies (Chiba, 1992; Chiba *et al*., 1994)^28,29^.

Consequently, we concluded that the lumbosacral plexus root changes with the thoracolumbar trade-off reported by studies of experimental embryology were not evident in humans. In comparison with other mammals, humans have a shorter lowest rib, which may be insufficient as a segmental compartment. For this reason, anastomoses between adjacent nerves form easily, potentially making it more difficult to evaluate the cranio–caudal shift of nerve roots.

To identify the phenotypic changes to the human lumbar plexus with different thoracolumbar counts, we used our results and those of previous reports on the transformation of lumbar nerves to compare spines with a reduced thoracolumbar count of 16 to those with an increased thoracolumbar count of 18 (Table 1 and Fig. 4). With the increasing vertebrae by two segments from 16 to 18, the nerve roots of the lumbar plexus tend to shift one segment caudally based on total 27 sides of 16 thoracolumbar vertebrae and 9 sides of 18 thoracolumbar vertebrae (Fig. 5A and Table 1). Thus, the change not be identifiable when comparing spines with the normal thoracolumbar count of 17 with those with a single abnormal vertebral mutation, as seen in conventional reports.

**Table 1:** Comparison data on analyzed nerve branches of lumbar plexus in 16 qnd 18 thoracolumbar vertebrae.

16 thoracolumbar vertebrae								18 thoracolumbar vertebrae						
		Bardeen	Horwitz	Ohuti	Morikawa	Present	Total			Bardeen	Horwitz	Morikawa	Present	Total
		(1902)	(1939)	(1951)	(1971)	study				(1902)	(1939)	(1971)	study	
Nerve	Origin	5 sides	6 sides	2 sides	6 sides	8 sides	27 sides	Nerve	Origin	2 sides	3 sides	2 sides	2 sides	9 sides
**lH**	19–20th	0		0	1	2	3/21 (14.3%)	**lH**	19–20th	0		0	0	0/6 (0.0%)
	20th	5		0	1	6	12/21 (57.1%)		20th	0		0	0	0/6 (0.0%)
	20–21st	0		2	1	0	3/21 (14.3%)		20–21st	0		1	0	1/6 (16.7%)
	21st	0		0	1	0	1/21 (4.8%)		21st	2		1	0	3/6 (50.%)
	21–22nd	0		0	0	0	0/21 (0.0%)		21–22nd	0		0	1	1/6 (16.7%)
	22nd	0		0	0	0	0/21 (0.0%)		22nd	0		0	1	1/6 (16.7%)
	unknown		6				NA, 6 sides		unknown		3			NA, 3 sides
**II**	20th	2		0	1	4	7/20 (35.0%)	**II**	20th	0		0	0	0/6 (0.0%)
	20–21st	2		0	2	1	5/20 (25.0%)		20–21st	0		0	0	0/6 (0.0%)
	21st	1		2	0	3	6/20 (30.0%)		21st	2		2	0	4/6 (66.7%)
	22nd	0		0	0	0	0/20 (0.0%)		22nd	0		0	2	2/6 (33.3%)
	unknown		6		1		NA, 7 sides		unknown		3			NA, 3 sides
**GF**	20–21st	2						**GF**	20–21st	0				
	21st	3							21st	0				
	21–22nd	0							21–22nd	1				
	22nd	0							22nd	1				
(no distinction)								(no distinction)						
**GFg**	20th			0	0	1	1/16 (6.3%)	**GFg**	20th			0	0	0/3 (0.0%)
	20–21st			0	1	1	2/16 (12.5%)		20–21st			0	0	0/3 (0.0%)
	21st			1	0	4	5/16 (31.3%)		21st			0	0	0/3 (0.0%)
	21–22nd			1	1	2	4/16 (25.0%)		21–22nd			1	0	1/3 (33.3%)
	22nd			0	0	0	0/16 (0.0%)		22nd			0	1	1/3 (33.3%)
	22–23nd			0	0	0	0/16 (0.0%)		22–23nd			0	1	1/3 (33.3%)
	unknown	5	6		2		NA, 11 sides		unknown	2	3	1		NA, 6 sides
**GFf**	20th			0	0	2	2/13 (15.4%)	**GFf**	20th			0	0	0/3 (0.0%)
	21st			1	0	3	4/13 (30.8%)		21st			0	0	0/3 (0.0%)
	21–22nd			1	0	2	3/13 (23.1%)		21–22nd			1	0	1/3 (33.3%)
	22–23rd				1		1/13 (7.7%)		22–23rd			0	2	2/3 (66.7%)
	unknown	5	6		3	1	NA, 14 sides		unknown	2	3	1		NA, 6 sides
**LST**	23rd			2	0	2	4/22 (18.2%)	**LST**	23rd		0	0	0	0/7 (0.0%)
	24th		6	0	4	6	16/22 (72.7%)		24th		0	0	0	0/7 (0.0%)
	25th		0	0	0	0	0/22 (0.0%)		25th		3	2	2	7/7 (100.0%)
	unknown	5					NA, 5 sides		unknown	2				NA, 2 sides

In developmental biology, there is a concept of positional information^41^, which refers to cells having information about their relative position within a cell population and changing their positions based on this. This has been used as a pattern formation model for chick limbs. A similar viewpoint has been applied in some macroscopic anatomical studies of humans, including ones that have investigated the lumbosacral plexus and the position of the sacroiliac joint^29^, the brachial plexus and the points where it penetrates the axillary artery branches^42,43^, and the composition of the sacral plexus and the penetration points of the internal iliac artery branches^44^. Interestingly, the positions of the lumbosacral and pudendal plexuses relative to the sacrum have been investigated and compared in various vertebrates by counting presacral vertebrae from the sacrum reversely as presacral numbers such as first presacral vertebra as last lumbar vertebra^45–48^. In the present study, the LST was relatively consistently the 24th spinal nerve, regardless of the thoracolumbar count or the presence of trade-off. This suggested that positional information about it being near the sacroiliac joint might have an influence. Based on this concept, we reexamined counting the nerve roots from the sacrum as a presacral number. Unfortunately, this reconsideration did not achieve the expected result (Fig. 5B).

Alternatively, the comparative anatomical and phylogenetic characteristics of vertebral variability and constraint may obtain reasonable evidence for the complex changes in nerve roots of the human lumbar plexus associated with vertebral mutations. To test this hypothesis, we reviewed comparative osteological data on the thoracolumbar formula and its proportion among mammals.

According to the various mammalian vertebral formulas in previous reports, the thoracolumbar count in non-human mammals tends to be fixed at 19 or 20 vertebrae^49,50^. Asher *et al*.^51^ analyzed the vertebral variability and constraint among individuals of representative species and between clades of mammals. Their data clarified that representing monotremes, xenarthrans, afrotherians, and primates show relatively high variation in thoracolumbar vertebral count, whereas rodents show low variability. These studies analyzed a small sample size of each of the mammalian representative species in order to understand the patterns of vertebral variation across mammals, and similar tendencies are also confirmed using large sample sizes of a single species (high variation in thoracolumbar count in spider monkeys^52^; stable 19 or 20 thoracolumbar count in rabbits^53^). Moreover, Williams (2011) found high levels of inter- and intraspecific variation among all hominoids except humans and eastern gorillas^54^. The common findings are that the humans exhibit a stable human thoracolumbar count of 17, as shown in our current study (94.6%) and that reduced vertebral counts such as 17 thoracolumbar and 5 lumbar vertebrae or less, are observed in only hominoids, bats, and some xenarthrans among the diversified mammals^49,50^.

In our previous study, we confirmed that the rodents exhibited low variation of the thoracolumbar count, but that some aerodynamic mammals showed higher variability in thoracolumbar count than other locomotive modes in rodents and their relatives^50^. For example, all 14 wild terrestrial brown rats examined had 13 thoracic and 6 lumbar vertebrae with no mutations and no thoracolumbar trade-off (Fig. 6A), whereas the 18 flying squirrels examined had variable thoracolumbar counts of 19 (78%), 20 (11%), and 21 (11%) with thoracolumbar trade-off (Fig. 6B).

Chang and Ruch (1947) also reported extraordinary thoracolumbar counts in spider monkeys, i.e., 7C–13T–5L–3S in 6/10 and 7C–14T–4L–3S in 4/10 cases. In addition to the supporting data on high variation in primate thoracolumbar count, interestingly, the spider monkey is a brachiator, i.e., has specialized locomotion. Locomotor behaviors such as suspension, climbing, clinging, and bridging between branches, are believed to be associated with a more stable and less flexible vertebral column (for details see Granatosky *et al*.^55^).

However, other data negates the hypothesis that specialized locomotor behaviors may relate to vertebral morphology. Li *et al*., (2018), for example, investigated thoracolumbar formula in 468 Kazakh sheep, which is a typical terrestrial mammal, but found high variation in thoracolumbar count; 18 (0.64%), 19 (75.63%), 20 (23.02%), and 21 (0.21%)^56^.

In the various evolutionary and functional implications of the thoracolumbar count, there is the evidence that a very stable thoracolumbar count of 19 in experimental rodents such as rats and mice, and humans are one of the exceptional mammals that reduced 17 thoracolumbar and 5 lumbar count. This evidence helps us to understand the different concomitant changes of the nerve roots in experimental rodents and humans.

The functional implications of the thoracolumbar vertebral proportion have also been discussed. Sargis (2001) explained that arboreal tupaiids are more thoracic vertebrae for increased stability on trees, but terrestrial tupaiids are more lumbar vertebrae for increased flexibility^57^. Our previous study showed that longer thoracic and shorter lumbar vertebrae were typically found in the examined terrestrial and arboreal rodents and relatives, but some gliding rodents did not share this trait (Fig. 6A,B). Similarly, the locomotion-dependent vertebral proportions of squirrels also showed that elongated lumbar vertebrae in gliding squirrels contribute to increased stiffness and are useful for stability and control during gliding compared to non-gliding squirrels^58,59^. In our measurement data, the total length of the lumbar vertebrae relative to the total length of the thoracolumbar vertebrae in most typical vertebral formulae of terrestrial rats, flying squirrels, and humans were 44.1% ± 3.2% (n = 14), 52.6% ± 1.7% (n = 12), and 35.2% ± 1.3% (n = 17), respectively. Therefore, our comparative osteology data indicates that the different locomotive modes in rodents may determine the thoracolumbar proportion and that humans have evidently shorter lumbar vertebrae than experimental rodents (Fig. 6).

However, there is an exceptional finding on the thoracolumbar proportions in Kazakh sheep^56^, which is a typical terrestrial mammal, but has a shorter relative length of the lumbar vertebrae (38.8%) in their typical thoracolumbar formula (13T–6L).

Therefore, we cannot find evidence for a functional and/or phylogenetic relationship with the thoracolumbar formula and proportion across mammals; therefore, it may be challenging to explain if the previous evidence in developmental biology can be applied to other mammalian taxa.

Interestingly, some differences were also found between the lumbar plexuses themselves in experimental rodents and humans. The lumbar plexuses of mice^24^ and rats^60^ are relatively simple, with few communications and segmental overlaps between the lumbar nerve roots, whereas the human lumbar plexus is more complex due to each nerve tending to be derived from multiple segments and ramified branches. In other words, differences in human lumbar nerve roots are harder to evaluate and less evident than those in experimental rodents.

In conclusion, we cannot provide evidence for the phylogenetic differences of the concomitant changes of nerve roots with vertebra across mammals, but we recognize the following differences in thoracolumbar vertebra and lumbar nerves limited to experimental rodents and humans: (1) Consistent 19 thoracolumbar count in experimental rodents such as rats and mice may produce highly reproducible and stable experimental results for nerve roots. (2) Conversely, reduction in thoracolumbar count in humans and shorter relative length of the human lumbar vertebrae than those in rodents may obscure concomitant changes of lumbar nerve roots with thoracolumbar mutations. (3) Regarding the more complicated human lumbar plexus, changes in the human lumbar nerve roots are harder to evaluate and less evident than in experimental rodents.

Therefore, the concomitant transformation of vertebrae and lumbar nerves reported in experimental rodents may not be evident in humans as a phenotypical morphology by the interaction between reduced thoracolumbar and lumbar counts, shorter vertebrae, and more complicated lumbar plexus itself in humans than those in experimental rodents.

## Methods

### Vertebral identification

Previous morphological criteria were followed for the identification of each vertebra in humans in the current study^50,51,57^.

Thoracic vertebrae were identified as those elements with articulated ribs in cadavers, or the presence of rib facets on or near the vertebral neural arch in dry specimens. Lumbar vertebrae were identified as those elements cranial to the sacro-iliac articulation lacking conspicuous rib facets and showing a transverse process regardless of length. In other words, the most important criterion for distinguishing between thoracic and lumbar vertebrae is whether they have articulated ribs. Sacral vertebrae were identified as those elements caudal to the vertebra participating in the sacro-iliac joint and one sacrum.

### Dissection

In total, 222 sides of 111 human cadavers were examined in this study. All donors and their families signed informed consent forms to formally donate bodies to Toho University School of Medicine for anatomical education and research uses. All the specimens were fixed with 7% formaldehyde solution administered via the radial or femoral artery and were preserved in 10% alcohol for more than 4 months. For 79 of the cadavers, the vertebral formula was estimated at the time of thoracotomy and laparotomy, with the exact vertebral count confirmed after making dry skeletons for the cases with a high possibility of vertebral mutation. The other 32 cadavers were subjected to CT imaging before dissection, which confirmed the vertebral formula. The coccygeal bones were excluded from our definitions of normal or abnormal vertebral formulas because some cases did not have clear calcification, making an accurate assessment difficult.

**Figure 7:**
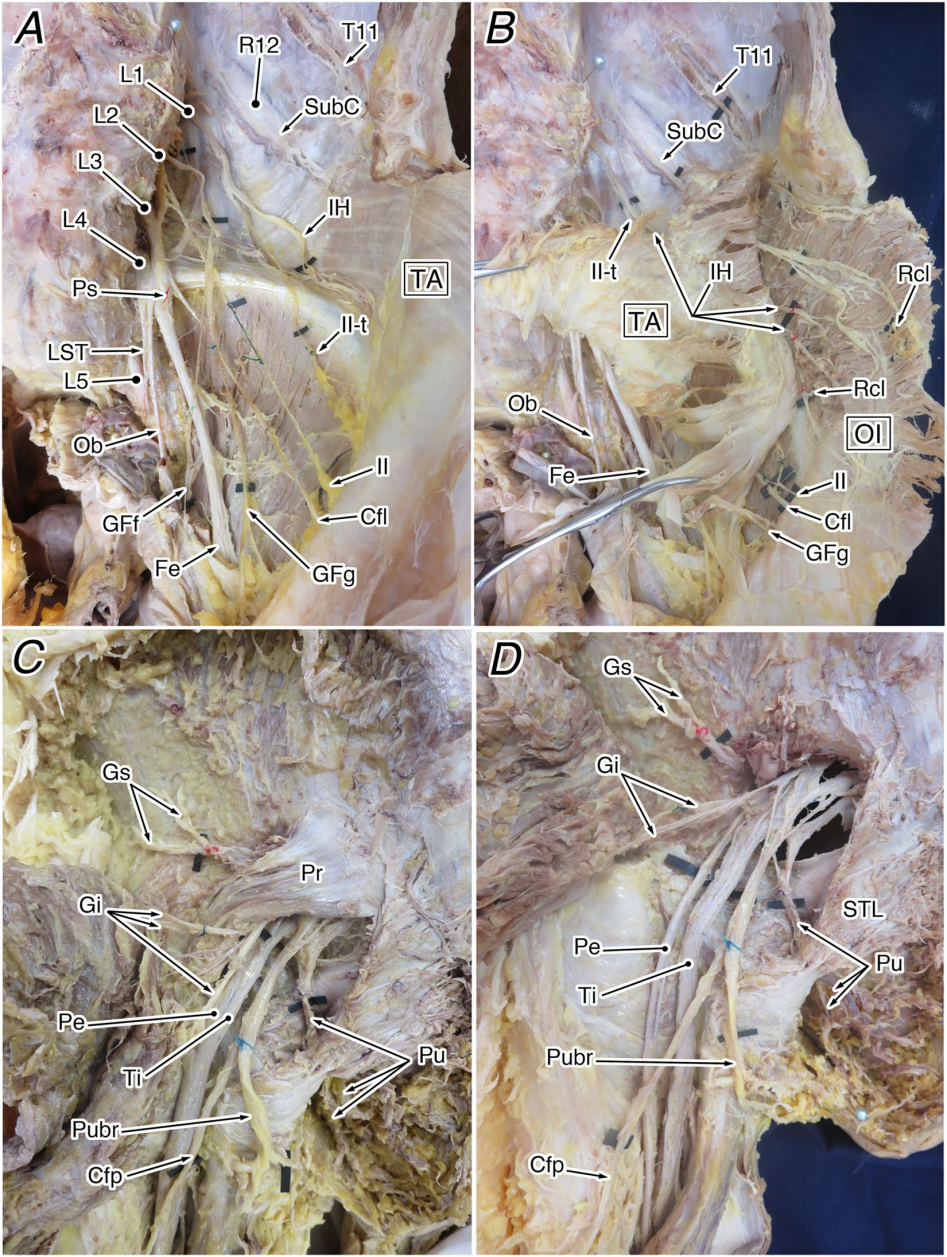
Steps in the dissection of the human lumbosacral plexus. (**A**,**B**) The lumbar plexus viewed from the ventral aspect. After confirming the origin and course of the nerves of the lumbar plexus (**A**), the intramuscular nerve course and distribution were examined (**B**). (**C**,**D**) The sacral plexus viewed from the dorsal aspect. After exposing the pelvic outlet and examining the relationship between the piriformis muscle and sacral plexus (**C**), the nerve roots of the sacral plexus were reconfirmed (**D**). Abbreviations: Cfl, lateral femoral cutaneous nerve; Cfp, posterior femoral cutaneous nerve; Fe, femoral nerve; GFf, femoral branch of the genitofemoral nerve; GFg, genital branch of the genitofemoral nerve; Gi, inferior gluteal nerve; Gs, superior gluteal nerve; IH. iliohypogastric nerve; II, ilioinguinal nerve; II-t, transitional ilioinguinal nerve; LST, lumbosacral trunk; Ob, obturator nerve; OI, obliquus internus muscle; Pe, common peroneal nerve; Pr, piriformis muscle; Ps, branch to the psoas major muscle; Pu, pudendal nerve; Pubr, pudendal branch of the posterior femoral cutaneous nerve; R12, 12th rib; Rcl, lateral cutaneous branch; STL, sacrotuberous ligament; SubC, subcostal nerve; TA, transversus abdominis muscle; Ti, tibial nerve.

After examination of the vertebral count, a detailed physical dissection of the lumbosacral plexus was performed, focusing on cases of thoracolumbar trade-off and abnormal thoracolumbar counts. The dissection was performed from the surface skin, in the usual manner, and the lumbosacral plexus was exposed. The spatial relationship between the lumbosacral plexus and the three layers of abdominal muscles was carefully examined, confirming its course and peripheral distribution (Fig. 7). The findings were recorded with a digital camera (IXY digital 620F; Canon, Tokyo, Japan) and detailed sketches.

The protocol for this study was reviewed and approved by ethics committee of Toho University Faculty of Medicine (reference number: A18015). All of the work conformed with the provisions of the 1995 Declaration of Helsinki (revised in Edinburgh in 2000).

### Imaging analysis

To confirm the vertebral formula and establish the three-dimensional topography of the spine, 32 cadavers were subjected to CT imaging with a Somatom Emotion 16 scanner (Siemens Healthcare GmbH, Erlangen, Germany), with the following parameters: tube voltage, 130 kV; current, 80–140 mA; slice width, 0.75 mm; and reconstruction width, 0.4–0.6 mm, depending on the body size. Axial DICOM images were reconstructed using commercial software (ZioCube ver. 1.0.0.4; Ziosoft Inc., Tokyo, Japan).

### Morphometry of dry skeletons

To determine the comparative anatomical characteristics of human vertebrae, every vertebra of 17 dry Asian unarticulated skeletons was measured using digital calipers (CD-20AXW; Mitsutoyo Co., Kawasaki, Japan). The results were compared with our previous data on the vertebral formulas of 14 wild brown rats (*Rattus norvegicus*) as an example of a general experimental animal and of 18 wild American flying squirrels (*Glaucomys volans*) as an example of a mammal with specialized locomotion (gliding)^50^.

### Terminology for the nerves

The controversial nerves from the abdominal wall to the lower limbs, known as the “border nerves” (Bardeen, 1902)^31^, were identified as follows. The *iliohypogastric nerve* (IH) was identified as the lowest nerve that ramifies the lateral cutaneous branch (Rcl) among the nerves similar to the subcostal nerve. The *ilioinguinal nerves* (II) were identified as nerves passing between the obliquus abdominis muscles but which did not ramify the Rcl. Because there were generally several II nerves, the one that entered the inguinal canal was regarded as the true illioinguinal nerve (II) and the others, including those with a transitional superficial course between the external and internal oblique muscles (referred to as “Rcas” by Kumaki (1981, 1994)), were considered to be transitional illioinguinal nerves (II-t). The *genitofemoral nerves* (GF) are transitional nerves of the lower limb type that usually penetrate the psoas muscle. Of these, the GF distributing to the anterior thigh along the external iliac vessels was identified as the femoral branch (GFf), and the other branches, including those entering the inguinal canal, were identified as the genital branches (GFg).

In spinal nerve counts, referring to “thoracic” and “lumbar” nerves can lead to confusion when considering vertebral trade-off cases or cases with abnormal vertebral counts. This reason, the serial number was used as the first spinal nerve from the first cervical nerve.

### Study limitations

Thoracolumbar formulas and counts have been difficult to obtain, despite searching for these over several years. Referring to past reports, it is clear its difficulties and so the trend was investigated including past reports. It will be necessary to collect further data in future studies. Nerve fiber analysis is necessary for complicated anastomoses that comprise more than three nerve segments, which we intend to investigate in our next study.

## Data Availability

The datasets used in this study are available from the corresponding author on reasonable request.
